# Supplementary material for: Blood transcriptomics analysis offers insights into variant-specific immune response to SARS-CoV-2
Source: Sci Rep. 2024 Feb 2;14:2808. doi: 10.1038/s41598-024-53117-w (PMC10837437; doi:10.1038/s41598-024-53117-w)
Supplement: Supplementary file 1 — Supplementary Information. [file 41598_2024_53117_MOESM1_ESM.pdf]

Supplemental Materials: **Blood transcriptomics analysis offers insights into variant-specific immune response to SARS-CoV-2**

Markus Hoffmann[1,2,3,*,**], Lina-Liv Willruth[1,*], Alexander Dietrich[1,*], Hye Kyung Lee[3], Ludwig Knabl[4], Nico Trummer[1], Jan Baumbach[5,6], Priscilla A. Furth[2,3,7], Lothar Hennighausen[2,3], and Markus List[1,**]

[1] Data Science in Systems Biomedicine, TUM School of Life Sciences, Technical University of Munich, Freising, Germany
[2] Institute for Advanced Study (Lichtenbergstrasse 2 a, D-85748 Garching, Germany), Technical University of Munich, Germany
[3] National Institute of Diabetes, Digestive, and Kidney Diseases, Bethesda, MD 20892, United States of America
[4] TyrolPath Obrist Brunhuber GMBH, Zams, Austria
[5] Chair of Computational Systems Biology, University of Hamburg, Hamburg, Germany
[6] Computational BioMedicine Lab, University of Southern Denmark, Odense, Denmark
[7] Departments of Oncology & Medicine, Georgetown University, Washington, DC, United States of America
*The authors wish it to be known that, in their opinion, the first three authors should be regarded as Joint First Authors.
** corresponding authors: markus.hoffmann@nih.gov; markus.list@tum.de

Abstract

Bulk RNA sequencing (RNA-seq) of blood is typically used for gene expression analysis in biomedical research but is still rarely used in clinical practice. In this study, we propose that RNA-seq should be considered a diagnostic tool, as it offers not only insights into aberrant gene expression and splicing but also delivers additional readouts on immune cell type composition as well as B-cell and T-cell receptor (BCR/TCR) repertoires. We demonstrate that RNA-seq offers insights into a patient's immune status via integrative analysis of RNA-seq data from patients infected with various SARS-CoV-2 variants (in total 196 samples with up to 200 million reads sequencing depth). We compare the results of computational cell-type deconvolution methods (e.g., MCP-counter, xCell, EPIC, quanTIseq) to complete blood count data, the current gold standard in clinical practice. We observe varying levels of lymphocyte depletion and significant differences in neutrophil levels between SARS-CoV-2 variants. Additionally, we identify B and T cell receptor (BCR/TCR) sequences using the tools MiXCR and TRUST4 to show that - combined with sequence alignments and BLASTp - they could be used to classify a patient's disease. Finally, we investigated the sequencing depth required for such analyses and concluded that 10 million reads per sample is sufficient. In conclusion, our study reveals that computational cell-type deconvolution and BCR/TCR methods using bulk RNA-seq analyses can supplement missing CBC data and offer insights into immune responses, disease severity, and pathogen-specific immunity, all achievable with a sequencing depth of 10 million reads per sample.

Supplemental Materials 1: Immune deconvolution methodology

MCP-counter and xCell are marker-gene-based cell-type deconvolution methods that leverage cell-type-specific marker genes to compute an enrichment score that can be compared across samples. Using gene expression data as input, MCP-counter computes scores for ten different cell types[1], whereas xCell computes scores for 64 different cell types[2].

The other two methods we use, EPIC[3] and quanTIseq[4], consider the deconvolution problem as a set of equations based on $b = C \times p$ (1), where b represents the gene expression matrix, C is a signature matrix containing a reference gene expression profile for all cell types and p, the vector denoting the proportions of each cell type, which are estimated. Both methods come with precomputed signature matrices within the immunedeconv package.

Each of the four methods gives results for various cell types. For example, xCell identifies 64 specialized cell types, while EPIC focuses on just six broader categories. To compare them fairly, we used a provided cell-type mapping in the immunedeconv package that translates the cell types into a standard language, which is called "controlled vocabulary cell types" that map specific cell types to broader cell types, e.g., mapping regulatory T cells to CD4+ T cells and allow a side-by-side comparison across methods.

Supplemental Materials 2: BCR/TCR repertoire methodology

MiXCR aligns sample reads to a customized database from GeneBank[5], groups them into clonotypes (i.e., receptor sequences that target the same antigen), corrects for polymerase chain reaction and sequencing errors, and exports the results in a tab-delimited file. TRUST4, in contrast, isolates candidate reads and carries out de novo assembly (i.e., this means it constructs longer sequences, or "contigs", from short reads based solely on their overlapping segments, without relying on any reference sequence). Then, it annotates the assembled contigs by aligning them to the ImmunoGeneTics database[6] and reports matched CDR3 sequences (i.e., unique areas of the antigen receptor gene that play a crucial role in the diversity of the immune response by determining the specific antigen-binding affinity of T and B cell receptors[7]).

Supplemental Materials 3: ClustalW algorithm in the BCR/TCR repertoire methodology

We use the ClustalW algorithm[8] to perform multiple sequence alignment via the *calc_dist_mat()* method from the AlignmentDistanceCalculator class in scirpy and the BLOSUM62[9] similarity matrix. Using the R package igraph[10] and the computed distances, we constructed a graph with sequences as nodes and their similarities represented by edges. This graph allowed visualization of method overlap by coloring nodes based on the reconstructing method that identified the sequence (either one of MiXCR and TRUST4 or both) and pinpointed unique sequences specific to SARS-CoV-2 variants by spotlighting sequences exclusive to infected samples and without connection to sequences from seronegative samples. According to the scirpy documentation, a sensible sequence distance cutoff is below 15, though we adopted a stricter threshold of 10, suggesting CDR3 sequences below this limit likely target the same antigen. For further analysis, we generated multiple sequence alignments of the reconstructed BCR and TCR CDR3 sequences using the msa R package[11] and depicted sequence conservation and regions with higher variation through sequence logos crafted with the ggseqlogo R package[12].

Supplemental Materials 4: Definition of positive and negative cases in sequence overlap comparison between sequencing depths

We defined the 'ground truth positive cases' as the set of sequences that we identified at full sequencing depth and are exclusively found in infected samples, meaning these sequences are absent in Seronegative samples and not similar to any sequence found in Seronegative samples. Conversely, the 'ground truth negative cases' consist of all other sequences that were detected but are also present in Seronegative samples. Similarly, we categorized the sequences identified at lower sequencing depths as positive cases if they are absent in Seronegative samples and not similar to any sequence in Seronegative samples, and the remaining sequences as the sets of negative cases that we test against the ground truth. Here, positive or negative cases do not denote whether a sequence is actually virus-specific but merely if it is a candidate for such a sequence.

Supplemental Table 1: Overview of the number of samples

Variant \ time	N/A	Day 0-5	Day 6-10	Day 11-15	Day 16-30	> Day 30	total
Alpha	-	18	12	10	17	10	67
Alpha EK	-	8	7	4	4	7	30
Gamma	-	1	1	1	-	-	3
Omicron BA.1	-	50	5	23	1	-	79
Omicron BA.2	-	13	-	5	5	-	23
Seronegative	50	-	-	-	-	-	50
total	50	90	25	43	27	17	252

Supplemental Table 1a: Table of the total number of samples per time point in the GEO database.

Variant \ time	N/A	Day 0-5	Day 6-10	Day 11-15	Day 16-30	> Day 30	total
Alpha	-	16	11	8	17	9	61
Alpha EK	-	8	6	4	4	7	29
Gamma	-	1	1	1	-	-	3
Omicron BA.1	-	3	5	23	1	-	32
Omicron BA.2	-	12	-	5	5	-	22
Seronegative	50	-	-	-	-	-	47
total	50	86	23	41	47	16	196

Supplemental Table 1b: Table of the number of samples per time point used for downstream analysis after quality control.

Supplemental Table 2: Excluded sample due to insufficient quality

sample	reason(s)
ID43_1st	distance to other samples, distance on PCA plot
B.1.351-ID5_3rd	problem with STAR alignment, failed threshold of minimum mapped reads
B.1.351-ID1_3rd_2	problem with GC content, not normally distributed[13]
ID29_2nd_2	problem with GC content, not normally distributed[13]
ID34_2nd_2	problem with GC content, not normally distributed[13]
ID38_3rd_2	problem with GC content, not normally distributed[13]
ID53_2nd_2	problem with GC content, not normally distributed[13]
BNT_Aus_20_2	problem with GC content, not normally distributed, distance to other samples, gene counts distributed badly (peak on chr 21)
A_118_Asymptom	distance to other samples, distance on PCA plot
B.1.351.ID14_1st	first sampling at day -1
ID_38_2nd	high mitochondrial gene counts (>0.1)[14]
ID_38_3rd	high mitochondrial gene counts (>0.1)[14]
ID_38_1st	because ID38 has pancytopenia due to myelodysplastic syndrome: decrease in all three peripheral blood cell lines [15]
B_425_Seronegative	high mitochondrial gene counts (>0.1)[14]
B_436_Seronegative	high mitochondrial gene counts (>0.1)[14]
B_446_Seronegative	high mitochondrial gene counts (>0.1)[14]
SRR18922948_COVID-19-Omicron_BA-1	problem with GC content, not normally distributed[13]
SRR18922909_COVID-19-Omicron_BA-1	too many overrepresented sequences, distance to other samples
SRR18922902_COVID-19-Omicron_--	no classification if BA.1 or BA.2

Supplemental Table 2: List of excluded samples due to quality concerns.

Supplemental Table 3: Identified BCR and TCR repertoire sequences with no similarities to seronegative BCR and TCR repertoire sequences and their hits with BLASTp in samples up to 200M sequencing depth

Sequence	Hit	Hit position	E-value
**CYSTDSSGNHRGVF**	**anti-SARS-CoV-2 immunoglobulin lambda [Homo sapiens]**	**8**	**6e-05**
CNSRDSSGNHLGVF	immunoglobulin light chain junction region [Homo sapiens]	1	1e-05
CQSYDSSNVVF	anti-SARS-CoV-2 immunoglobulin light chain [Homo sapiens]	7	0.050
CMQGTHWPTF	immunoglobulin light chain junction region [Homo sapiens]	1	0.002
CQSYDSSLSGSGVF	immunoglobulin light chain junction region [Homo sapiens]	1	3e-05
CLQHDNFPYTF	immunoglobulin light chain junction region [Homo sapiens]	1	3e-04
CAAWDDSLNGHVVF	immunoglobulin light chain junction region [Homo sapiens]	1	1e-06
CLQHDNFPLTF	(11-NOV-2022) anti-SARS-CoV-2 immunoglobulin light chain [Homo sapiens] [Mus musculus]	5	0.010
CQAWDSSVVF	(15-MAR-2023) anti-SARS-CoV-2 immunoglobulin light chain [Homo sapiens]	4	0.27
CVVSDRGSTLGRLYF	T cell receptor alpha chain V region (clone 1V alpha 24-1) - human (fragment) [Homo sapiens]	1	8e-07
CSSYTSSSTVF	(15-FEB-2023) anti-SARS-CoV-2 immunoglobulin light chain variable region [Homo sapiens]	82	2.6
CQSYDSSLSGSYVF	immunoglobulin light chain junction region [Homo sapiens]	1	7e-06
CQAWDSSTVF	immunoglobulin light chain junction region [Homo sapiens]	1	0.037
CETWDSNTRVF	anti-SARS-CoV-2 spike protein immunoglobulin light chain variable region [Homo sapiens]	10	0.006
**CQQRSNWPPTWTF**	**anti-SARS-CoV-2 immunoglobulin light chain variable region [Homo sapiens]**	**8**	**5e-06**

Supplemental Table 3: The fifteen unique sequences that were distinct from healthy BcR and TCR repertoire samples with their first BLASTp result or the first result with SARS-CoV-2 connection, as well as the E-value and position of the result. Two sequences among the seven identified exhibit significant importance, as indicated by their notably low E-values. These values suggest the rarity of achieving a similar score by chance for these

sequences. The first sequence (CYSTDSSGNHRGVF), identified in a study by Graham et al.[16], was among over 100 mononuclear antibodies (mAbs) characterized for their interaction with epitopes from individuals infected with SARS-CoV-2. This study also demonstrated that some of these mAbs possess the ability to neutralize SARS-CoV-2. The second noteworthy sequence (CQQRSNWPPTWTF) emerged from a study by Jennewein et al. [17]. In this study, 198 antibodies were identified, with fourteen being distinguished as neutralizing antibodies (nAbs) against SARS-CoV-2. The study further explored how some of these nAbs can block the binding of ACE-2, thereby inhibiting viral entry into cells.

Supplemental Table 4: Identified BCR and TCR repertoire sequences with no similarities to seronegative BCR and TCR repertoire sequences and their hits with BLASTp in samples up to 10M sequencing depth

Sequence	Hit	Hit position	E-value
CAAWDDSLNGWVF	anti-SARS-CoV-2 immunoglobulin light chain [Homo sapiens]	61	4e-05
CAAWDDSLNGPVF	anti-SARS-CoV-2 immunoglobulin heavy chain variable region [Homo sapiens]	39	2e-04
CQSADSSGTYVVF	anti-SARS-CoV-2 immunoglobulin light chain [Homo sapiens]	13	0.002
CQSYDSSLSGSVF	anti-SARS-CoV-2 immunoglobulin light chain [Homo sapiens]	34	0.003
CGTWDSSLSAGVF	anti-SARS-CoV-2 immunoglobulin light chain [Homo sapiens]	35	0.001
CLQHNSYPWTF	anti-SARS-CoV-2 immunoglobulin light chain variable region [Homo sapiens]	28	0.002
CMQATQFPRTF	anti-SARS-CoV-2 immunoglobulin light chain [Homo sapiens]	3	0.007
CALWEVQELGKKIKVF	T-cell receptor gamma chain [Homo sapiens]	1	7e-09

Supplemental Table 4: The eight unique sequences that were distinct from healthy BcR and TCR repertoire samples derived from the MiXCR and TRUST4 results for downsampled results with a sequencing depth of 10 Mio. For each sequence, the first BLASTp result or the first result with SARS-CoV-2 connection is shown, as well as the E-value and position of the result.

Supplemental Figure 1a:

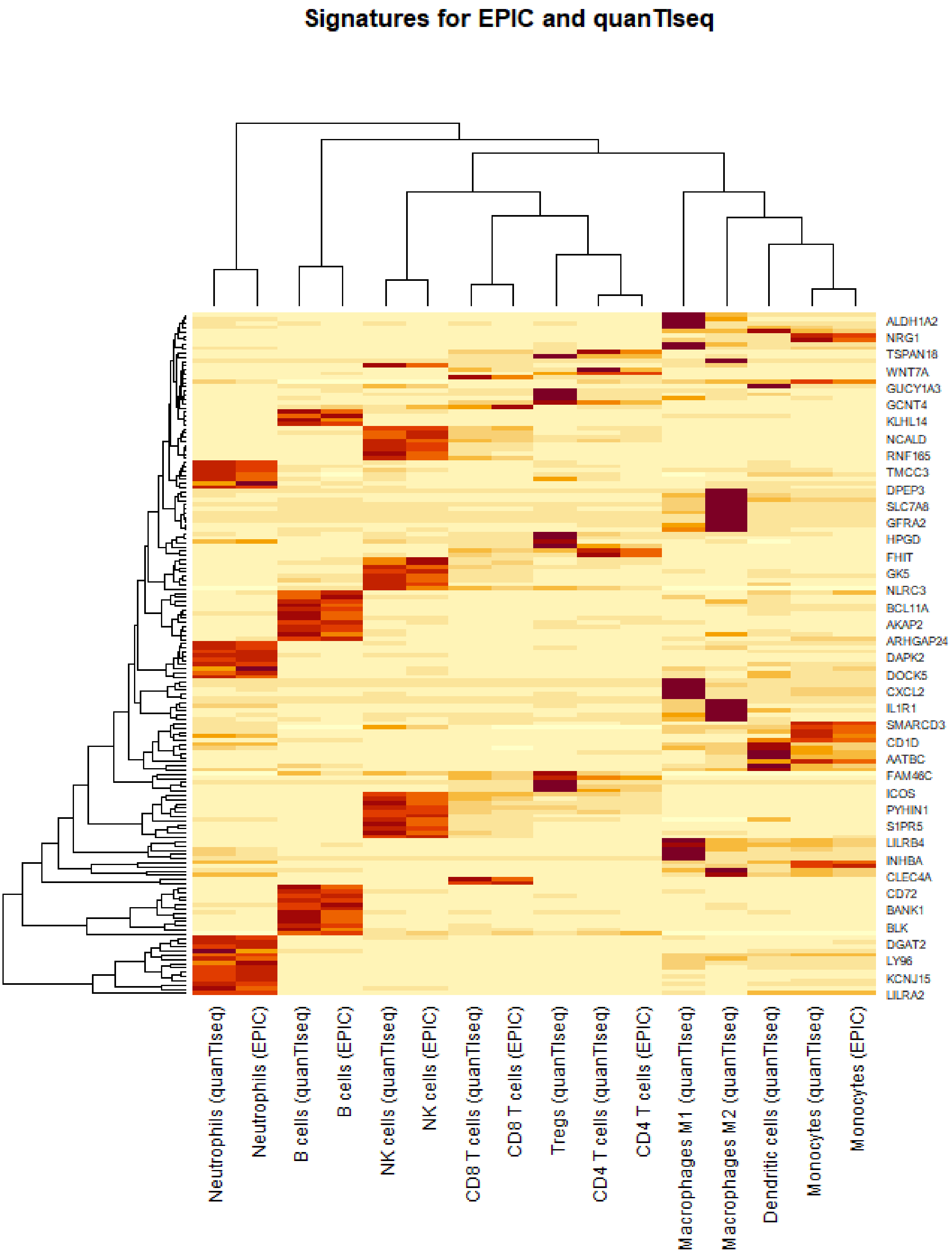

Supplemental Figure 1a: We visualized the marker genes shared by quanTIseq and EPIC.

Supplemental Figure 1b:

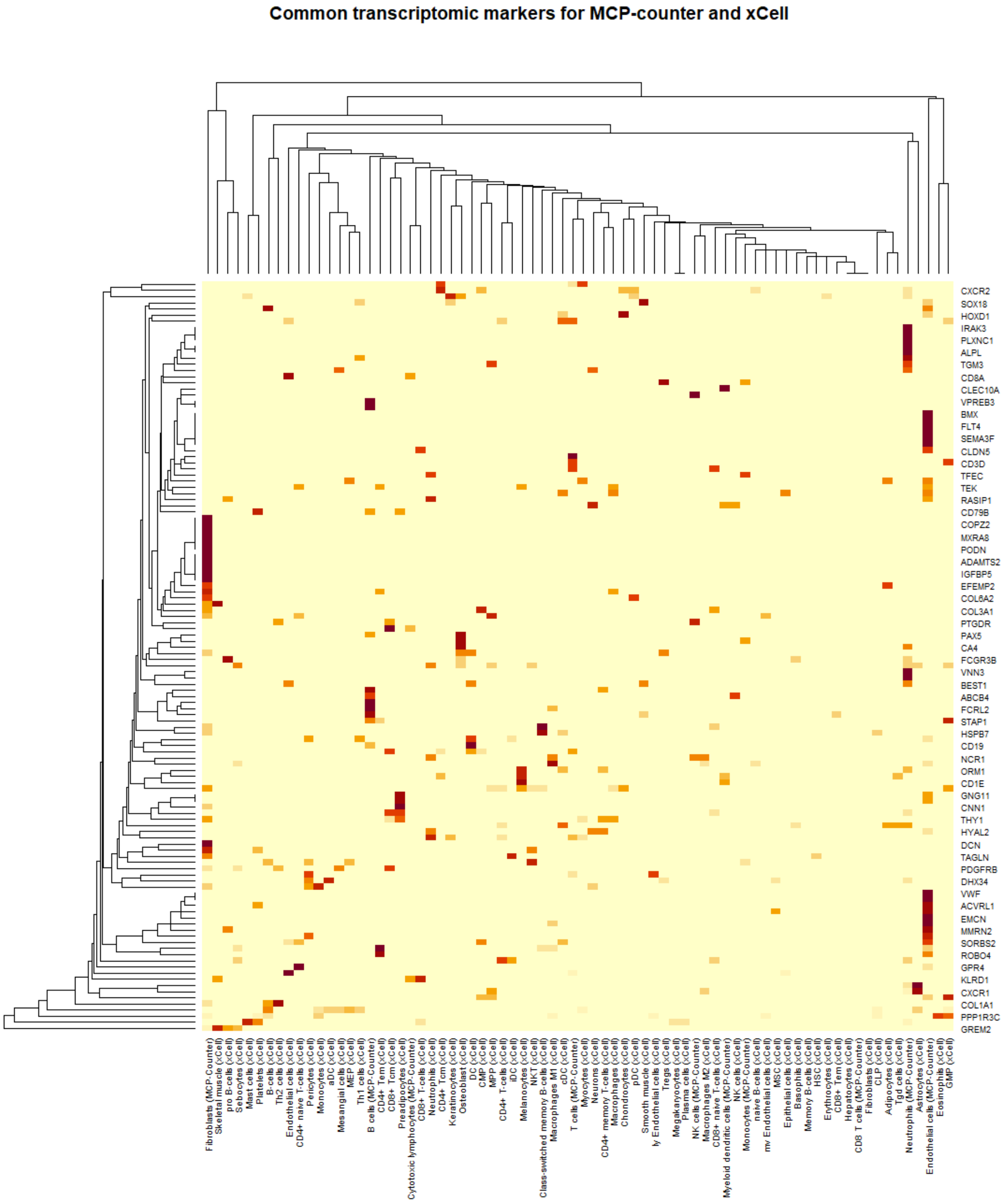

Supplemental Figure 1a: We visualized the marker genes shared by MCP-counter and xCell.

Supplemental Figure 2:

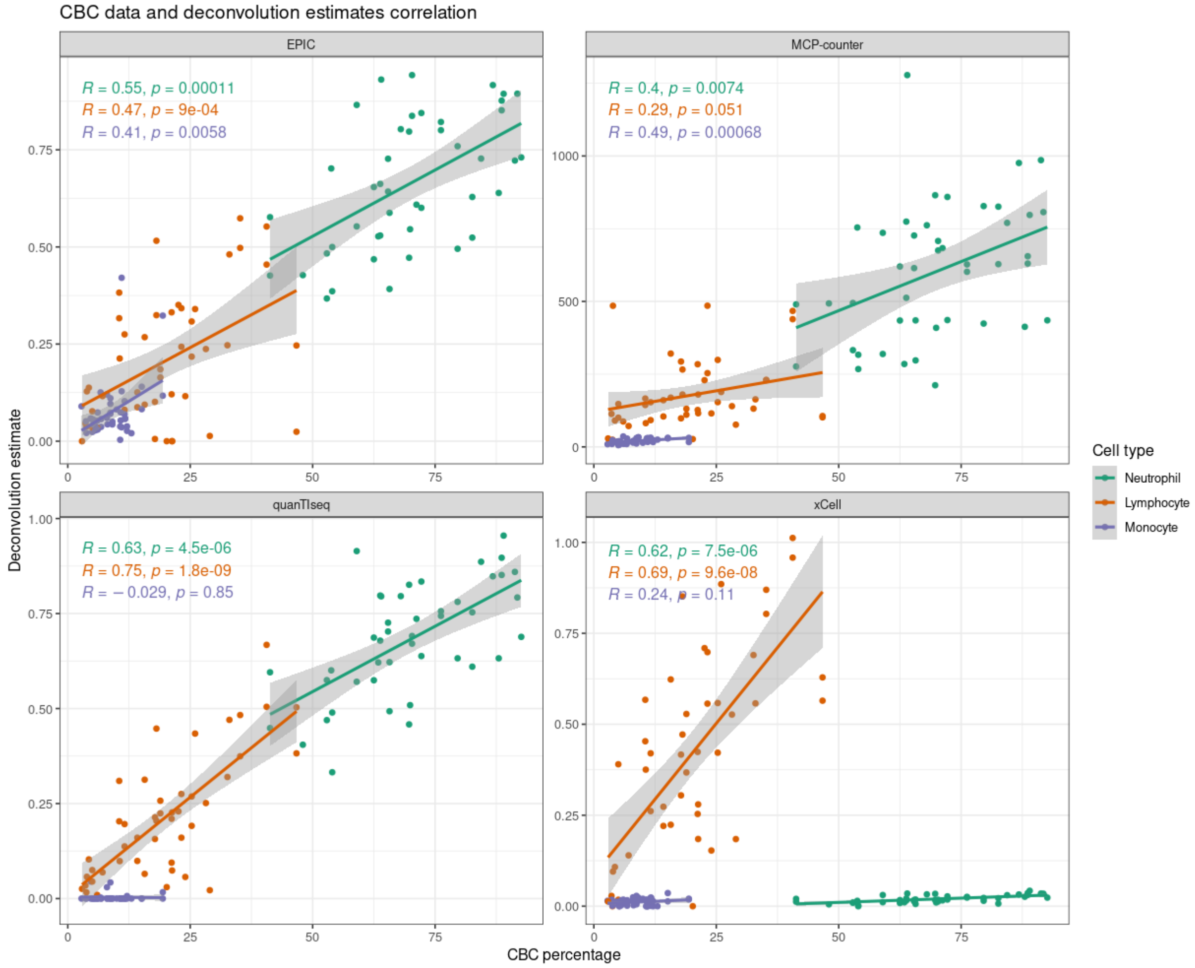

Supplemental Figure 2: Correlation between CBC data and immune deconvolution scores across lymphocytes, monocytes, and neutrophils using all four deconvolution methods. The colored lines display the linear regression model for each cell type; the shaded areas are the confidence intervals. Pearson correlation coefficients with p-values are given in addition.

Supplemental Figure 3: P-values of the slopes for the linear regressions of the immune deconvolution results over time.

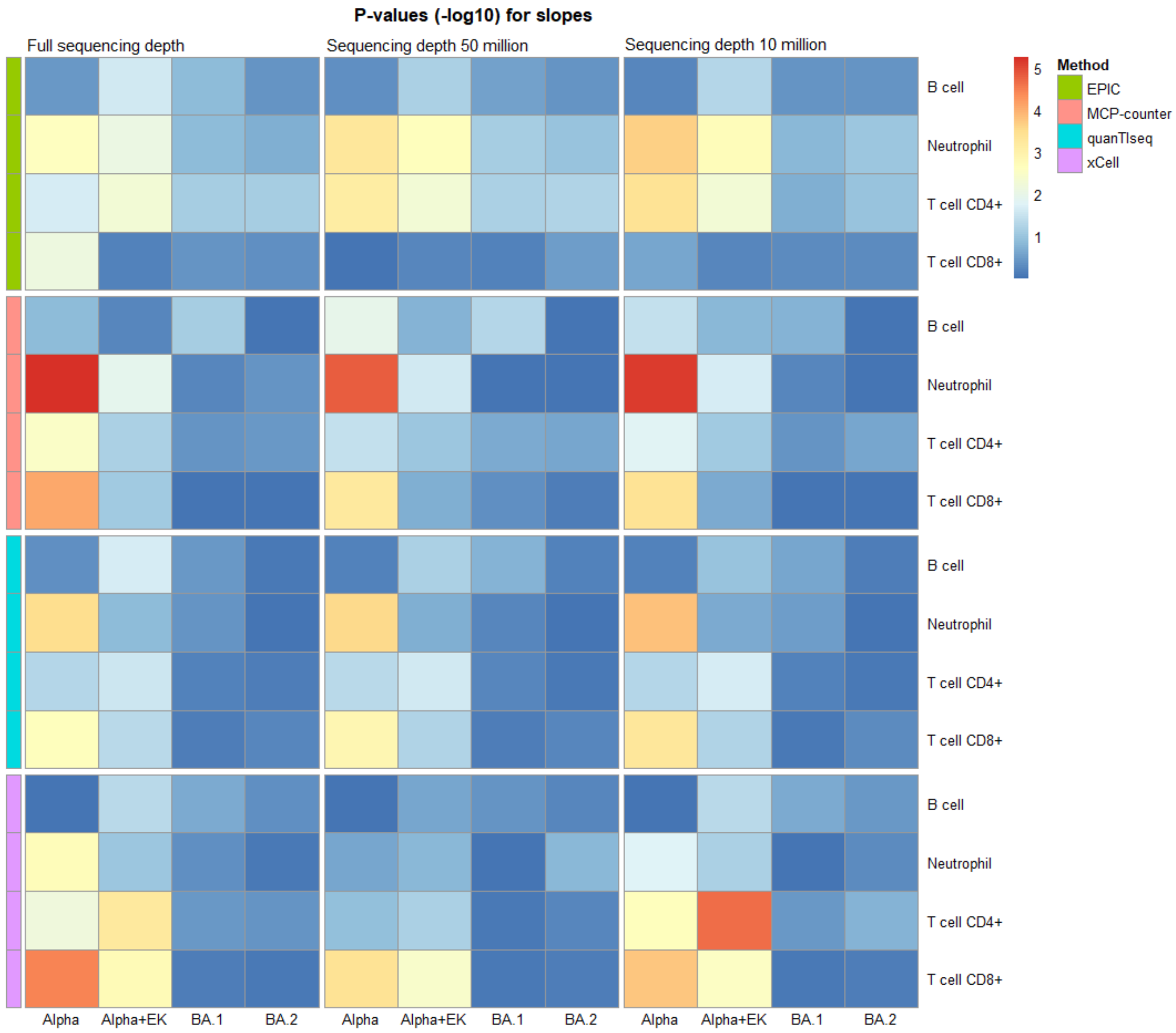

Supplemental Figure 3: The -log10 transformed P-values for the slopes of linear regressions performed in the timepoint analysis (Figure 3, Suppl. Figs. 6a and 6b) per method, cell type and COVID variant for full and both reduced sequencing depths.

Supplemental Figure 4:

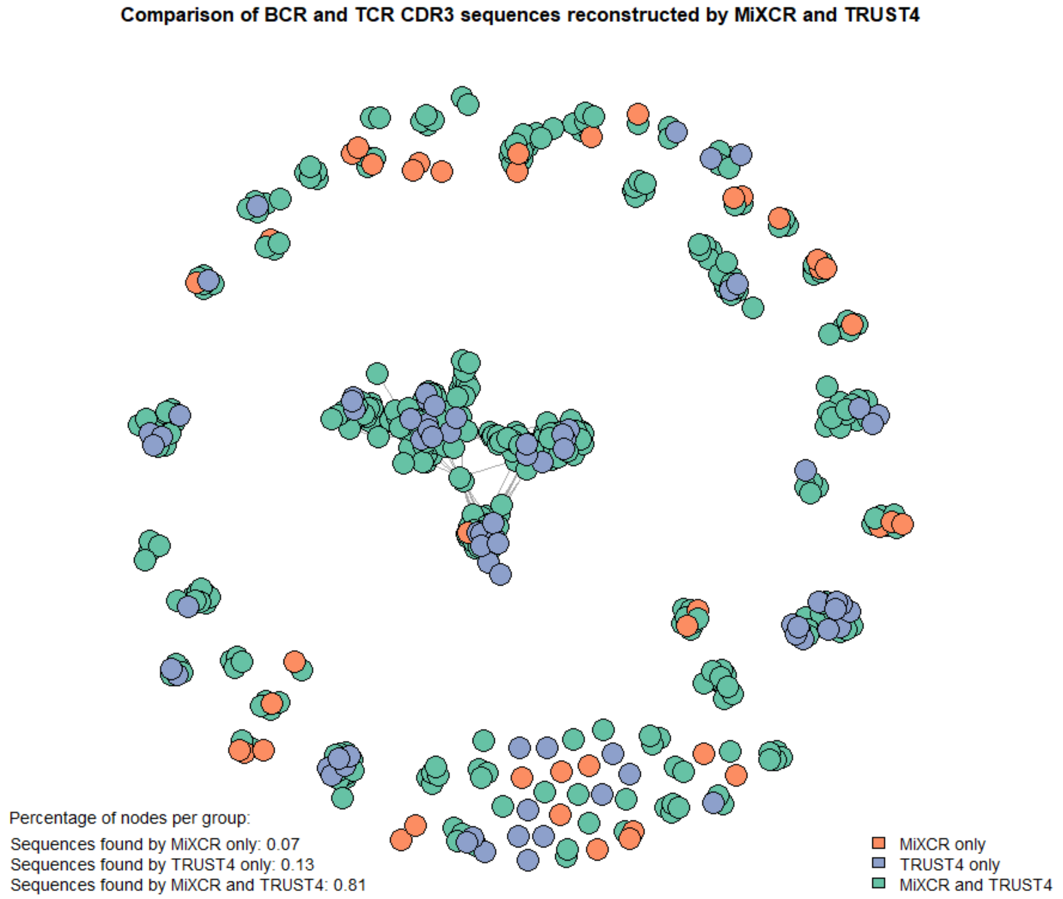

Supplemental Figure 4: We visualized the results of MiXCR and TRUST4 in a graph, where sequences are nodes, with an edge between two nodes if they are similar (see Materials and Methods). The sequences found by both tools are represented in green, those found only by MiXCR are in orange, and those found only by TRUST4 are in blue. The majority of nodes are green, confirming that most sequences were found by both tools.

Supplemental Figure 5:

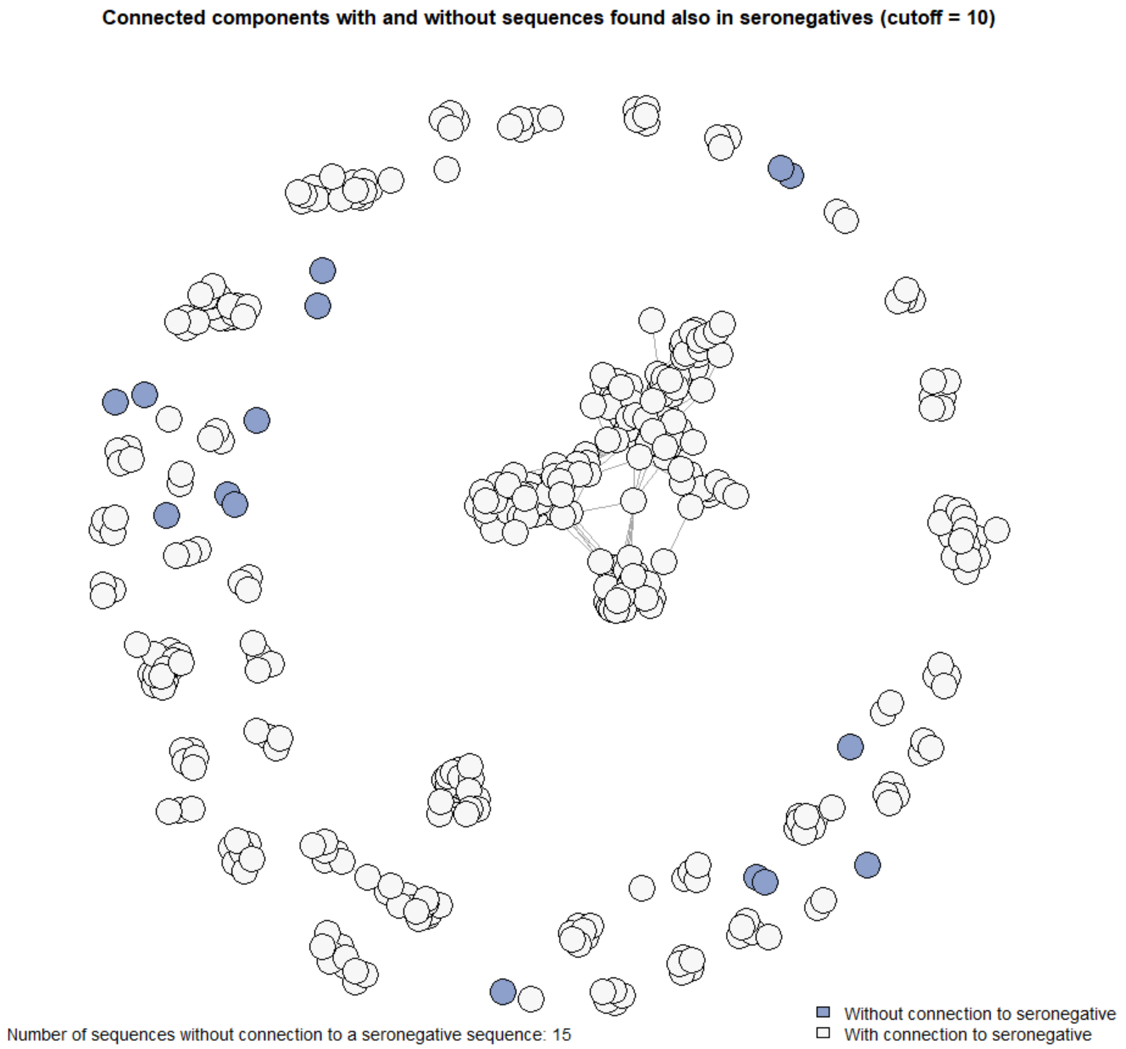

Supplemental Figure 5: MiXCR and TRUST4 both predicted 15 BCR/TCR repertoire sequences that were distinct from seronegative samples.

Supplemental Figure 6:

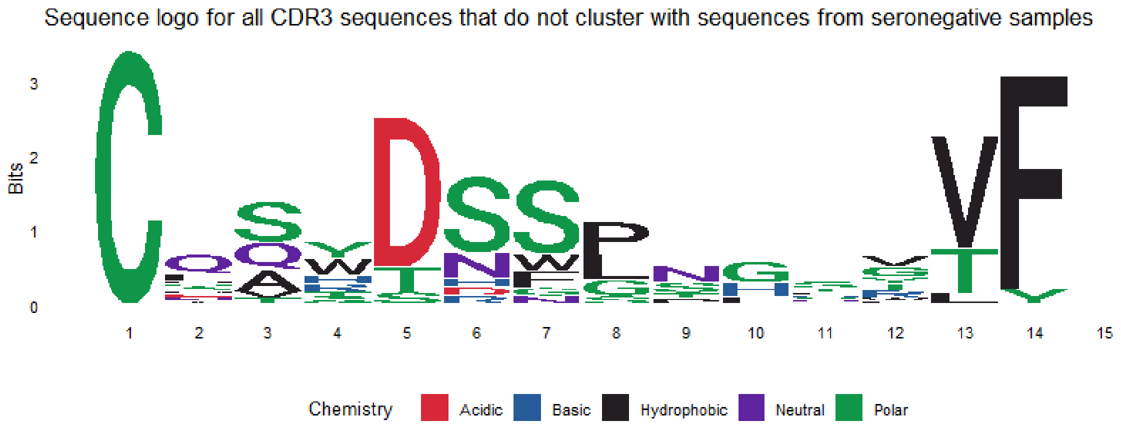

Supplemental Figure 6: Sequence logo of the BCR and TCR repertoire sequences that were not similar to the ones found in the Seronegative samples.

Supplemental Figure 7a:

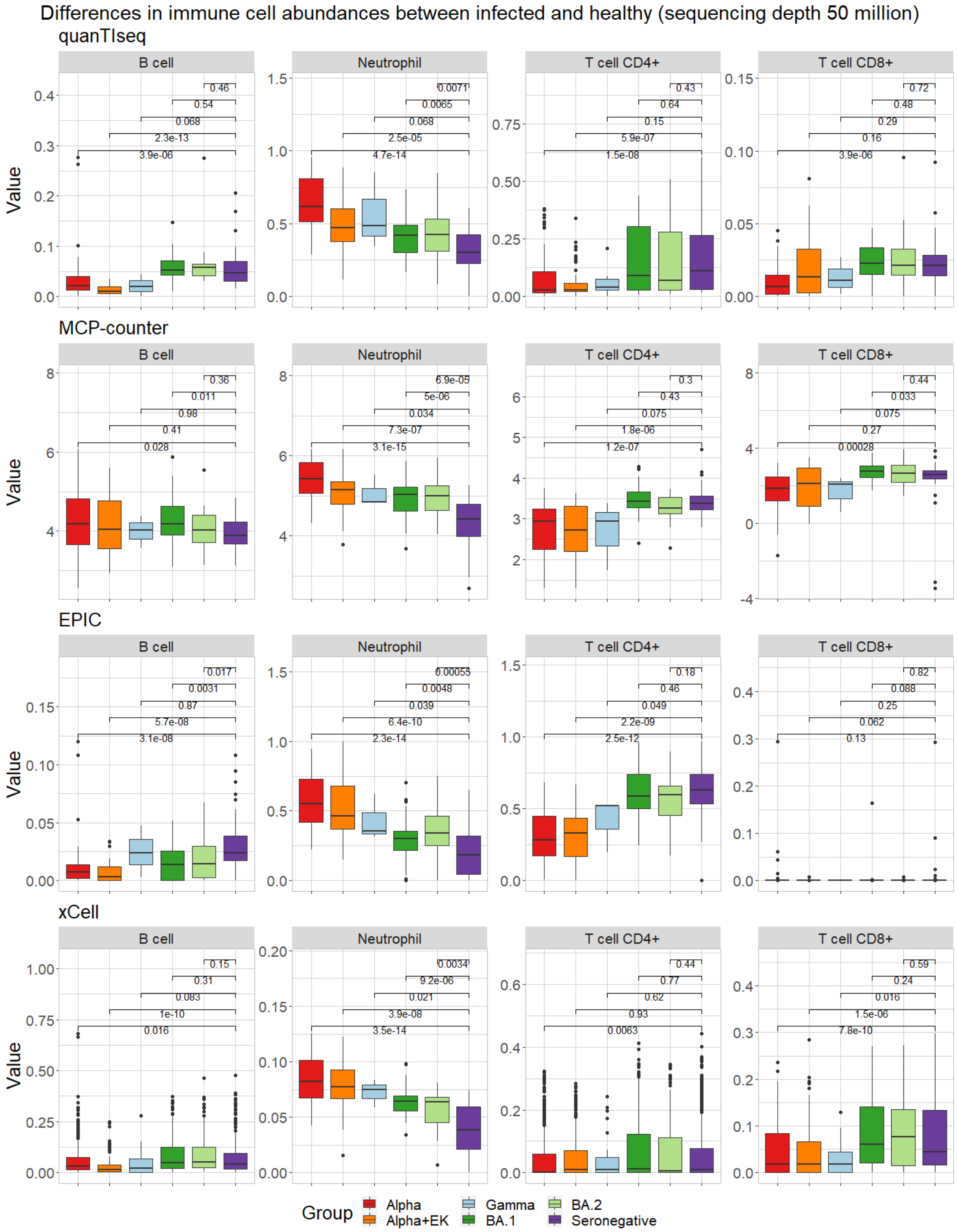

Supplemental Figure 7a: Immune deconvolution results for downsampled sequencing depth 50 million. The abundance of immune cells (given by percentage or method-specific score) detected by the immune deconvolution methods quanTIseq, MCP-counter, EPIC, and xCell over all time points combined for the immune cells B cell, Neutrophil, T cell CD4+, and T cell CD8+.

Supplemental Figure 7b:

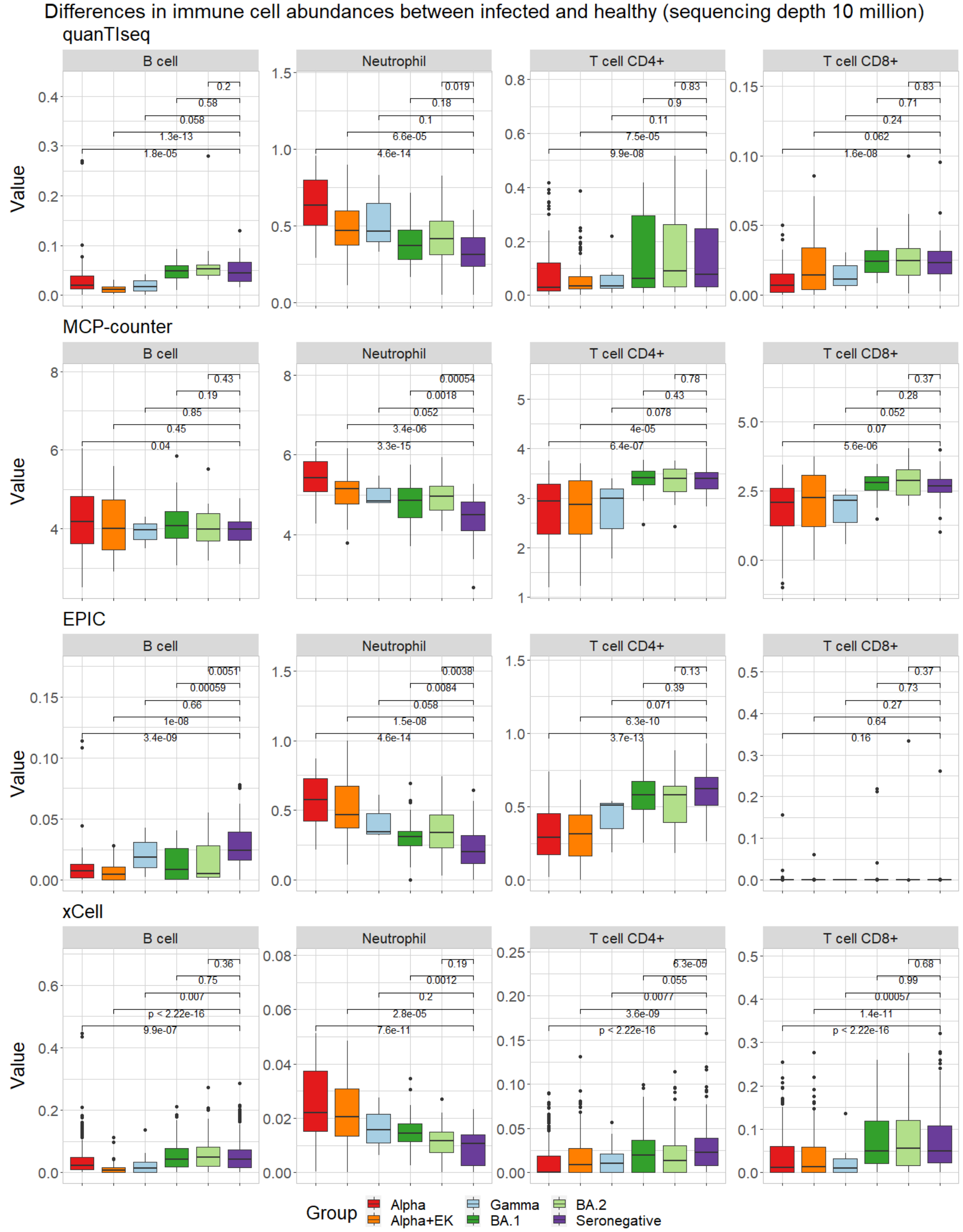

Supplemental Figure 7b: Immune deconvolution results for downsampled sequencing depth 10 million. The abundance of immune cells (given by percentage or method-specific score) detected by the immune deconvolution methods quanTIseq, MCP-counter, EPIC, and xCell over all time points combined for the immune cells B cell, Neutrophil, T cell CD4+, and T cell CD8+.

Supplemental Figure 8a:

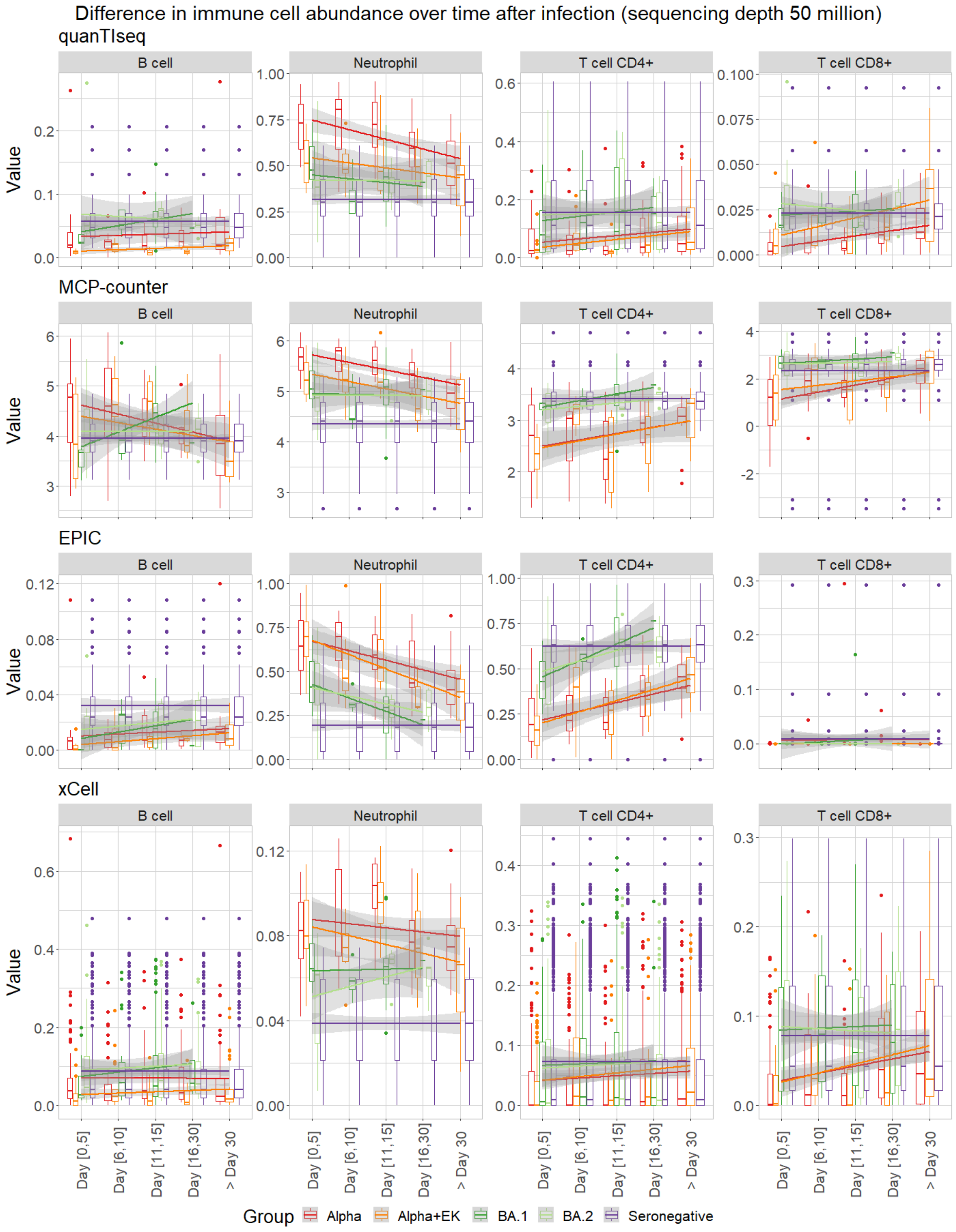

Supplemental Figure 8a: Immune deconvolution results for downsampled sequencing depth 10 million. Cell-type fractions separated over brackets 0-5, 6-10, 11-15, 16-30, and >30 days after hospitalization or onset of symptoms detected by the immune deconvolution methods quanTseq, MCP-counter, EPIC, and xCell for the immune cells B cell, Neutrophil, T cell CD4+, and T cell CD8+. The Gamma variant has been removed in this analysis due to poor sample size per time bracket (Suppl. Tables 1a-b).

Supplemental Figure 8b:

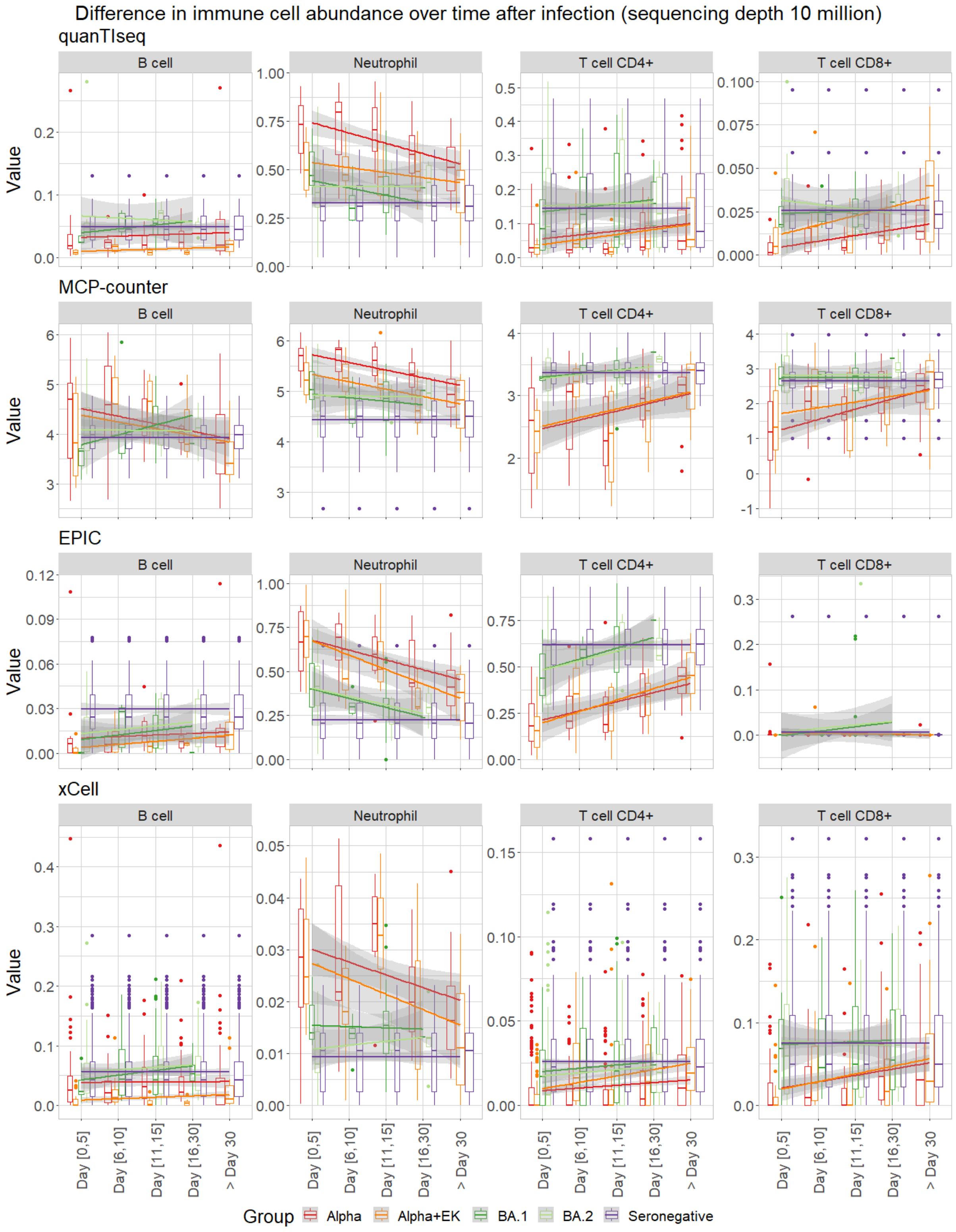

Supplemental Figure 8b: Immune deconvolution results for downsampled sequencing depth 10 million. Cell-type fractions separated over brackets 0-5, 6-10, 11-15, 16-30, and >30 days after hospitalization or onset of symptoms detected by the immune deconvolution methods quanTseq, MCP-counter, EPIC, and xCell for the immune cells B cell, Neutrophil, T cell CD4+, and T cell CD8+. The Gamma variant has been removed in this analysis due to poor sample size per time bracket (Suppl. Tables 1a-b).

Supplemental Figure 9: Pearson Correlation of CBCs and deconvolution estimates over different sequencing depths

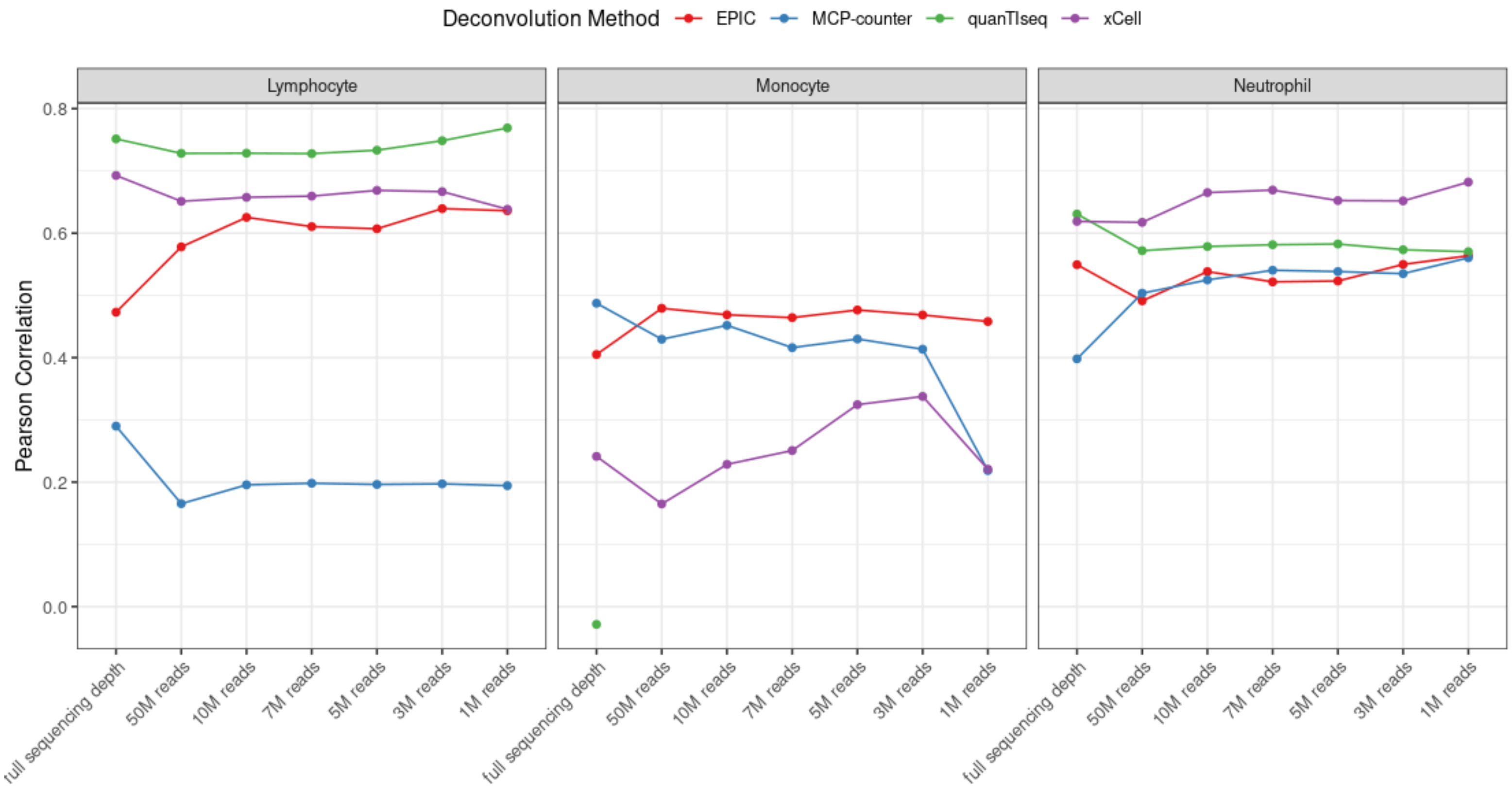

Supplemental Figure 9: Pearson Correlation coefficients for full sequencing depth (left-most point in each box) and subsequently downsampled datasets. Correlations are calculated separately for cell type and deconvolution method.

Supplemental Figure 10:

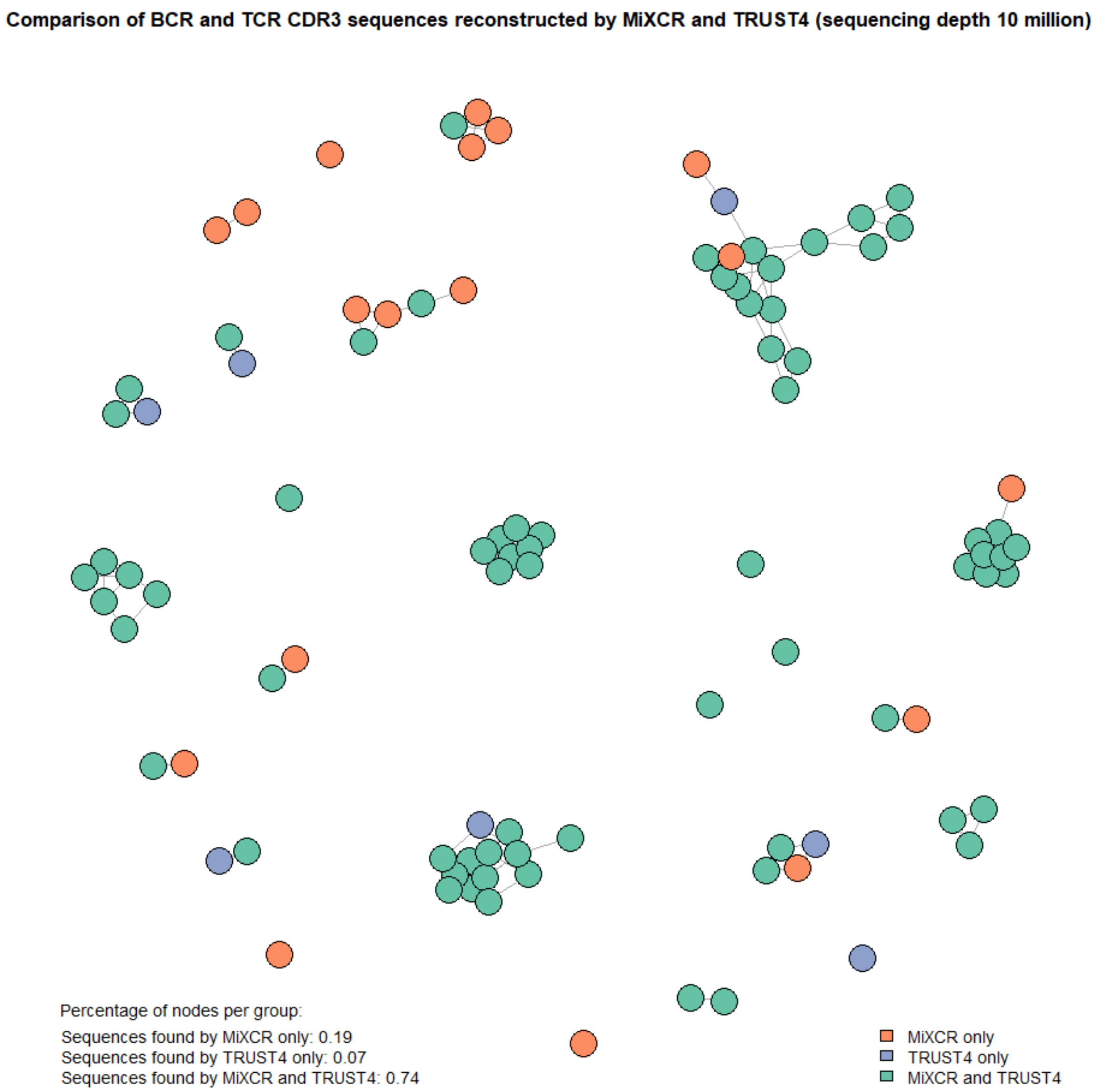

Supplemental Figure 10: Network displaying the BCR and TCR sequences reconstructed by MiXCR (orange) and TRUST (blue) and sequences found by both tools as nodes. Interactions (edges) between the sequences show sequence similarities. 74 % of unique sequences were found by both tools, 19 % were only found by MiXCR, and 7 % were only found by TRUST4.

Supplemental Figure 11:

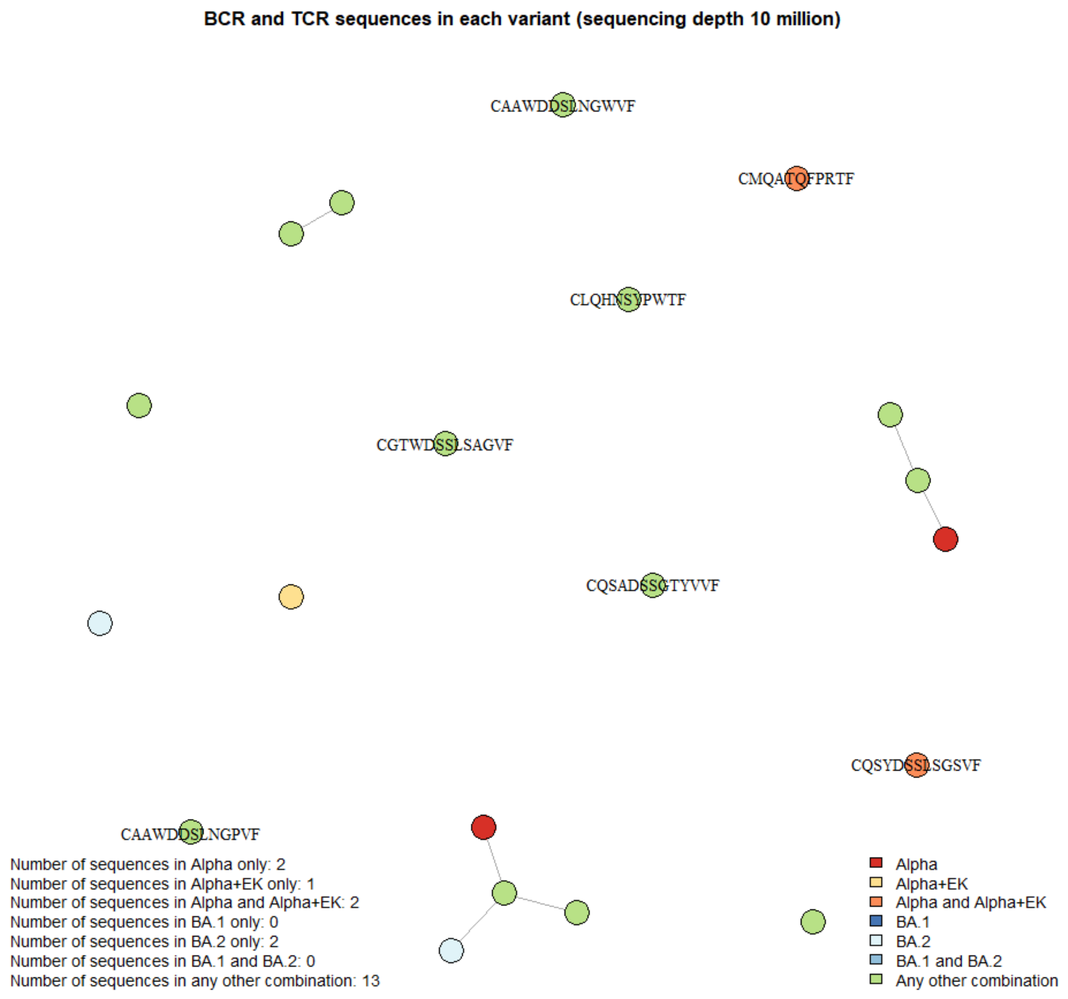

Supplemental Figure 11: Network showing sequences that are only found in infected samples as nodes and sequence similarities (score below 10) as edges. Annotated sequences are sequences with an anti-SARS-CoV-2 immunoglobulin pBLAST hit.

References

1. Becht, E. *et al.* Estimating the population abundance of tissue-infiltrating immune and stromal cell populations using gene expression. *Genome Biol.* **17**, 218 (2016).
2. Aran, D., Hu, Z. & Butte, A. J. xCell: digitally portraying the tissue cellular heterogeneity landscape. *Genome Biol.* **18**, 220 (2017).
3. Racle, J., de Jonge, K., Baumgaertner, P., Speiser, D. E. & Gfeller, D. Simultaneous enumeration of cancer and immune cell types from bulk tumor gene expression data. *Elife* **6**, (2017).
4. Finotello, F. *et al.* Molecular and pharmacological modulators of the tumor immune contexture revealed by deconvolution of RNA-seq data. *Genome Med.* **11**, 34 (2019).
5. Smakaj, E. *et al.* Benchmarking immunoinformatic tools for the analysis of antibody repertoire sequences. *Bioinformatics* **36**, 1731–1739 (2020).
6. Lefranc, M.-P. *et al.* IMGT, the international ImMunoGeneTics information system ®. *Nucleic Acids Res.* **33**, D593–D597 (2005).
7. Yu, K., Shi, J., Lu, D. & Yang, Q. Comparative analysis of CDR3 regions in paired human αβ CD8 T cells. *FEBS Open Bio* **9**, 1450–1459 (2019).
8. Thompson, J. D., Higgins, D. G. & Gibson, T. J. CLUSTAL W: improving the sensitivity of progressive multiple sequence alignment through sequence weighting, position-specific gap penalties and weight matrix choice. *Nucleic Acids Res.* **22**, 4673–4680 (1994).
9. Henikoff, S. & Henikoff, J. G. Amino acid substitution matrices from protein blocks. *Proc. Natl. Acad. Sci. U. S. A.* **89**, 10915–10919 (1992).
10. igraph – Network analysis software. https://igraph.org/.
11. Bodenhofer, U., Bonatesta, E., Horejš-Kainrath, C. & Hochreiter, S. msa: an R package for multiple sequence alignment. *Bioinformatics* btv494 Preprint at https://doi.org/10.1093/bioinformatics/btv494 (2015).
12. Wagih, O. ggseqlogo: a versatile R package for drawing sequence logos. *Bioinformatics*

33, 3645–3647 (2017).

13. Per Sequence GC Content. https://www.bioinformatics.babraham.ac.uk/projects/fastqc/Help/3%20Analysis%20Modules/5%20Per%20Sequence%20GC%20Content.html.
14. Lun, A. T. L., McCarthy, D. J. & Marioni, J. C. A step-by-step workflow for low-level analysis of single-cell RNA-seq data with Bioconductor. *F1000Res.* **5**, 2122 (2016).
15. Lancaster, I., Patel, D., Sethi, V., Connelly, W. & Namey, J. Myelodysplastic syndrome in a case of new-onset pancytopenia. *Clin. Case Rep.* **10**, e05533 (2022).
16. Graham, C. *et al.* Neutralization potency of monoclonal antibodies recognizing dominant and subdominant epitopes on SARS-CoV-2 Spike is impacted by the B.1.1.7 variant. *Immunity* **54**, 1276–1289.e6 (2021).
17. Jennewein, M. F. *et al.* Isolation and characterization of cross-neutralizing coronavirus antibodies from COVID-19+ subjects. *Cell Rep.* **36**, 109353 (2021).
